# Identification of Modulated MicroRNAs Associated with Breast Cancer, Diet, and Physical Activity

**DOI:** 10.3390/cancers12092555

**Published:** 2020-09-08

**Authors:** Luca Falzone, Maria Grimaldi, Egidio Celentano, Livia S. A. Augustin, Massimo Libra

**Affiliations:** 1 IRCCS Istituto Nazionale Tumori “Fondazione G. Pascale”, Epidemiology Unit, 80131 Naples, Italy; m.grimaldi@istitutotumori.na.it (M.G.); e.celentano@istitutotumori.na.it (E.C.); livia.augustin@utoronto.ca (L.S.A.A.); 2 Department of Biomedical and Biotechnological Sciences, University of Catania, 95123 Catania, Italy; 3 Research Centre for Prevention, Diagnosis, and Treatment of Cancer, University of Catania, 95123 Catania, Italy

**Keywords:** microRNA, breast cancer, biomarkers, bioinformatics, prognosis, epigenetic, diet, physical activity

## Abstract

**Simple Summary:**

Healthy diet and physical activity are able to induce beneficial molecular modifications that have been associated with a lower risk of breast cancer (BC) incidence and a better prognosis for BC patients. Although the beneficial effects of healthy lifestyle have been described, the beneficial epigenetic modifications induced by dietary and exercise intervention in BC patients have not been elucidated yet. On these bases, the aim of the present study was to computationally identify microRNAs (miRNAs) strictly associated with BC progression and with dietary and exercise interventions. Through several computational approaches, a set of miRNAs modulated by diet and exercise and useful as diagnostic and prognostic biomarkers for BC was identified. The results obtained represent the starting point for further validation analyses performed on BC patients undergoing lifestyle interventions to propose the miRNAs here identified as novel biomarkers for BC management.

**Abstract:**

*Background:* Several studies have shown that healthy lifestyles prevent the risk of breast cancer (BC) and are associated with better prognosis. It was hypothesized that lifestyle strategies induce microRNA (miRNA) modulation that, in turn, may lead to important epigenetic modifications. The identification of miRNAs associated with BC, diet, and physical activity may give further insights into the role played by lifestyle interventions and their efficacy for BC patients. To predict which miRNAs may be modulated by diet and physical activity in BC patients, the analyses of different miRNA expression datasets were performed. *Methods:* The GEO DataSets database was used to select miRNA expression datasets related to BC patients, dietary interventions, and physical exercise. Further bioinformatic approaches were used to establish the value of selected miRNAs in BC development and prognosis. *Results:* The analysis of datasets allowed the selection of modulated miRNAs associated with BC development, diet, and physical exercise. Seven miRNAs were also associated with the overall survival of BC patients. *Conclusions:* The identified miRNAs may play a role in the development of BC and may have a prognostic value in patients treated with integrative interventions including diet and physical activity. Validation of such modulated miRNAs on BC patients undergoing lifestyle strategies will be mandatory.

## 1. Introduction

Breast cancer (BC) represents the second most diagnosed cancer worldwide and the leading cause of cancer death in women accounting, respectively, for 2,088,849 million new diagnoses and over 625,000 deaths annually [1]. Despite the advancement of anticancer surgical and pharmacological treatments, both BC incidence and mortality rates have remained almost unchanged during the last 40 years [2,3,4].

Recently, several integrative therapies have been proposed for the treatment of BC patients in order to improve the efficacy of standard anticancer approaches, reduce chemotherapy side effects, ameliorate patient quality of life, and to reduce BC mortality [5,6,7]. Currently adopted integrative therapies for BC patients include nutraceutical products like vitamins, flavonoids and natural extracts [8,9], prebiotics and probiotics [10,11], the adoption of a healthy lifestyle mainly represented by moderate or intense physical exercise [12,13], and the adherence to healthy or hypocaloric diets [5,14].

Such approaches have been adopted following the association of cancer development with the dysbiosis of human microbiota, unhealthy lifestyle habits, hypercaloric diets, hormonal imbalances, and metabolic disorders [15,16,17,18,19]. In this context, it was demonstrated that sedentary lifestyle and dietary carbohydrates have detrimental effects for insulinemia and glycemia responsible for chronic inflammation and, consequently, with neoplastic transformation [20]. In particular, some foods, characterized by high glycemic index (GI) and high dietary inflammatory index (DII), are known to alter cellular homeostasis, inducing the over-expression of pro-inflammatory cytokines and hormones including Insulin-like Growth Factor 1 (IGF-1) [21,22]. All these pro-inflammatory stimuli and growth factors can lead to the alteration of key cellular processes and signaling transduction pathways like MAPKs and PI3K/Akt pathways, prompting the malignant transformation of cells [23,24]. Beyond these molecular changes that alter cellular homeostasis, dietary and environmental factors are also able to induce epigenetic modifications including changes in microRNA (miRNA) expression or in genetic methylation status [25,26,27,28].

Studies have shown that diet and physical exercise are able to modulate both DNA methylation status and miRNA expression levels and possibly BC risk [29,30]. Several lines of evidence have been accumulated in recent years about the protective role of diet against various human cancers including breast cancer. The “Prevención con Dieta Mediterránea” (PREDIMED) study demonstrated that adherence to the Mediterranean diet and olive oil intake is associated with a significant lower risk (68%) of BC incidence [31]. Other diets such as Okinawan and plant-based diets, low energy density, and low glycemic load dietary patterns are associated with BC prevention and better prognosis [32]. In the same manner, different studies have highlighted the protective role of exercise, which is able to reduce BC incidence and mortality inducing both genetic and epigenetic modulation [33,34,35].

All this evidence leads researchers to investigate the epigenetic modifications and beneficial effects induced by dietary restriction and physical exercise in women and BC patients to assess the reduction of BC risk, the decrease of BC recurrence, and the therapeutic potential of food administration [5,36,37]. However, no convincing data have been generated on this topic and no epigenetic biomarkers predictive of the therapeutic efficacy and patient prognosis have been identified as yet. The evaluation of the expression levels of miRNAs modulated by diet and exercise and directly correlated with BC progression and/or therapeutic efficacy may represent an additional strategy to establish patients prognosis, predict cancer recurrence, and evaluate the efficacy of such integrative treatments.

Therefore, the aim of the present study was to computationally select a set of miRNAs strictly involved in BC development and progression, but also effectively modulated by diet and exercise. For this purpose, the analysis and integration of different BC, diet, and exercise miRNAs expression profiling datasets obtained from the Gene Expression Omnibus DataSets (GEO DataSets; https://www.ncbi.nlm.nih.gov/gds) database was performed. In addition, further bioinformatic prediction analyses were performed on the selected miRNAs in order to establish their prognostic significance and clinical usefulness for BC patients.

This methodology represents the starting point for novel validation studies performed in BC patients treated with hypocaloric diets or physical activity interventions.

## 2. Results

### 2.1. miRNA Microarray Expression Profiling Dataset Selection

The search of miRNAs expression profiling performed on GEO DataSets allowed for the identification of several datasets containing miRNA expression related to BC, diet, and exercise. By using specific search terms, 229, 47, and 21 miRNA microarray expression profiling datasets were identified for BC, diet, and exercise, respectively. However, following the inclusion and exclusion criteria described in the “Materials and Methods” section, most of these datasets were excluded, thus selecting only eight datasets for BC, three datasets for diet, and eight datasets for exercise (Table 1). The majority of BC datasets were excluded from the analysis because they contained miRNA expression data obtained from circulating fluids or a limited number of samples, or in vitro or animal models. In the diet and exercise datasets, those containing samples derived from in vitro or animal studies were excluded in order to reduce variability between different species.

The selection of eight different datasets for BC allowed for the integrated evaluation of miRNA expression levels related to 881 BC samples and 187 normal breast tissue samples (control). In the same manner, the integrated analysis of three miRNA expression datasets related to dietary intervention was carried out on a total of 76 samples of which 38 were before and 38 after dietary intervention. Regarding exercise datasets, the computational analysis was performed on 226 samples of which 114 were before and 112 after physical activities.

### 2.2. Computational Identification of microRNAs Involved in Breast Cancer

**Figure 1:**
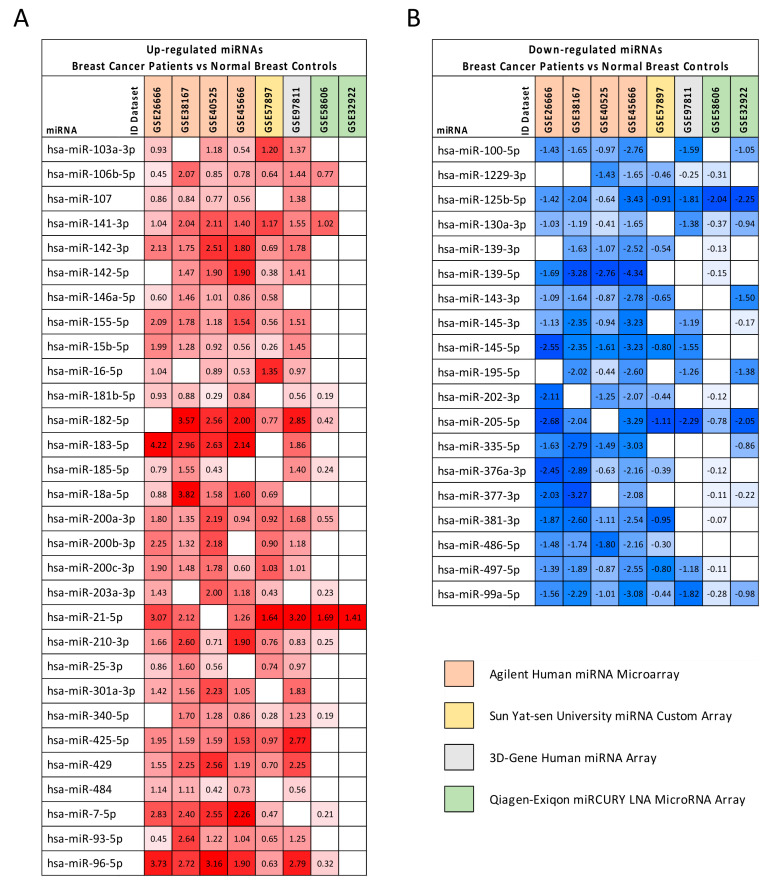
Differentially expressed miRNAs in BC samples compared to normal breast tissues in at least five out of eight datasets. The results of differential analyses were expressed as log2FC values indicating with red scale boxes the upregulated miRNAs (Panel (**A**)) and with blue scale boxes the downregulated ones (Panel (**B**)). Dataset IDs were marked with different colors depending on the platform technology used.

The differential analysis between BC samples and controls performed by using GEO2R allowed for the identification of a list of dysregulated miRNAs for each dataset. In total, a set of 49 miRNAs that were significantly dysregulated (*p* < 0.01) was found in at least five out of the eight datasets selected. Of these miRNAs, 30 were significantly upregulated in BC samples compared to the controls, while 19 were downregulated (Figure 1).

Among the upregulated miRNAs, those most commonly altered in the eight datasets and with the highest log2FC values were hsa-miR-106b-5p, hsa-miR-141-3p, hsa-miR-182-5p, hsa-miR-183-5p, hsa-miR-200 family, hsa-miR-21-5p, hsa-miR-7-5p, and hsa-miR-96-5p. Similarly, among the downregulated miRNAs, the most important were hsa-miR-125b-5p, hsa-miR-123 130a-3p, hsa-miR-139-5p, hsa-miR-205-5p, hsa-miR-497-5p, and hsa-miR-99a-5p. It is important to note that almost all of these miRNAs are known to be involved in several tumors, as will be argued in the Discussion section.

To further corroborate the results obtained from the analysis of the eight GEO DataSets miRNA expression datasets, differential analyses between the expression levels of the miRNAs in BC samples and normal control were performed on the Cancer Genome Atlas Breast Cancer (TCGA BRCA) dataset. As reported in Table 2, this analysis demonstrated that the upregulation and downregulation levels of the 49 significantly dysregulated miRNAs in BC were confirmed for almost all miRNAs selected (Table 2).

Of note, the most upregulated (e.g., hsa-miR-182-5p, hsa-miR-183-5p, hsa-miR-21-5p) and downregulated (e.g., hsa-miR-139-5p, hsa-miR-145-3p, hsa-miR-205-5p) miRNAs in the GEO DataSets microarray platforms were also strongly dysregulated in the TCGA BRCA database. In addition, among the 49 selected GEO DataSets miRNAs, three miRNAs (i.e., hsa-miR-146a-5p, hsa-miR-25-3p and miR-202-3p) showed no alteration of their expression in TCGA BRCA database, while the hsa-miR-1229-3p was downregulated in the GEO DataSets platforms and upregulated in TCGA BRCA database. Overall, these results further confirm the robustness of the computational analyses performed on the GEO DataSets for BC.

### 2.3. Computational Identification of microRNA Modulated by Diet and Exercise

**Figure 2:**
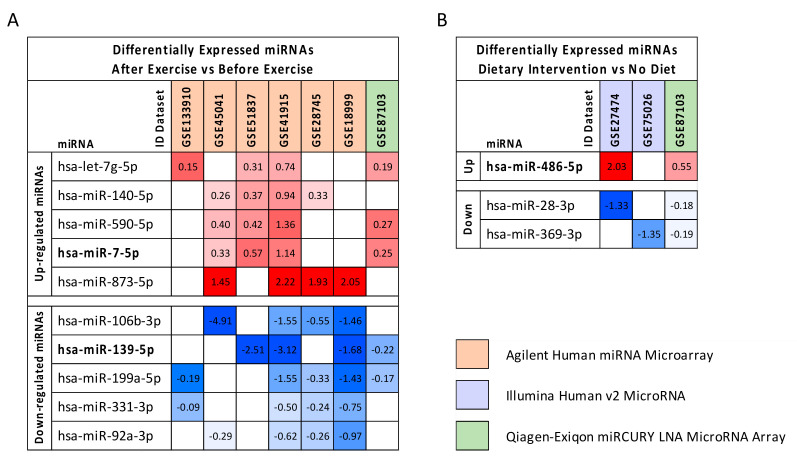
Panel (**A**): Differentially expressed miRNAs before and after exercise. Panel (**B**): Differentially expressed miRNAs before and after dietary interventions. miRNAs in common in at least >50% of the selected datasets were selected. The results of the differential analyses were expressed as log2FC values indicating with red scale boxes the upregulated miRNAs and blue scale boxes the downregulated ones. Dataset IDs were marked with different colors depending on the platform technology used. In bold are reported miRNAs also involved in BC.

As described for BC datasets, the GEO2R differential analyses performed for diet and exercise datasets revealed that these interventions may beneficially modulate some miRNAs. In particular, 10 dysregulated miRNAs were found when merging the seven exercise datasets selected. Of these, five were upregulated and five downregulated (Figure 2A).

In the same manner, three deregulated miRNAs, one upregulated and two downregulated, were identified by analyzing the three diet datasets alone (Figure 2B).

It is important to note that some of these miRNAs were also deregulated in BC. In particular, hsa-miR-486-5p and hsa-miR-7-5p were significantly upregulated by diet and exercise, respectively. These miRNAs were also downregulated and upregulated, respectively, in the BC dataset. Similarly, hsa-miR-139-5p was significantly downregulated by exercise and also downregulated in BC.

### 2.4. Breast Cancer (BC) miRNA Modulation of Epithelial-Mesenchymal Transition (EMT) Genes

**Figure 3:**
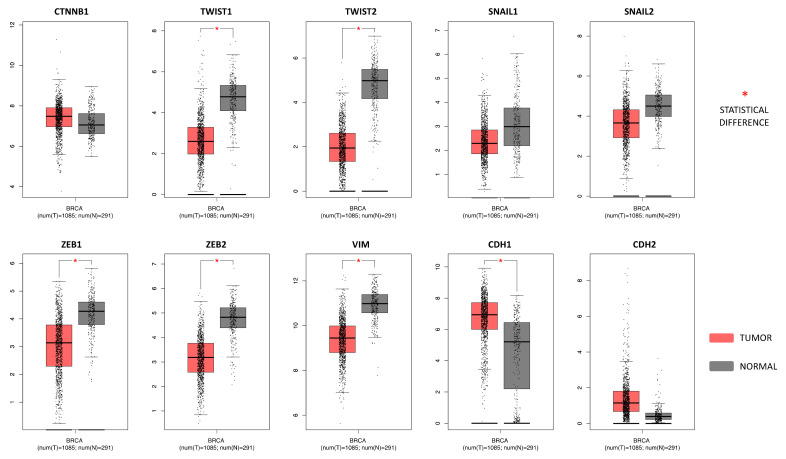
EMT gene expression levels in breast cancer samples compared to the controls. The GEPIA software performs a four-way ANOVA differential analysis (using sex, age, ethnicity, and disease state (tumor or normal) as variables for calculating differential expression. The *p*-values were adjusted according to the Benjamini and Hochberg false discovery rate. The *p*-value threshold was set at 0.01 (* = *p* < 0.01). The relative expression levels were first log2(TPM+1) transformed and the log2FC was defined as median (Tumor)—Median (Normal), where TPM is the transcript count per million.

As expected, the gene expression profiling interactive analysis (GEPIA) showed that the majority of epithelial-mesenchymal transition (EMT) genes were significantly deregulated in breast cancer samples compared to controls reflecting an epithelial phenotype. Overall, almost all the EMT genes analyzed were downregulated in breast cancer samples compared to normal breast tissues, except for CDH1, CTNNB1, and CDH2 that were upregulated in BC. However, of these, only CDH1 was statistically upregulated. On the contrary, significantly downregulated EMT genes in thee BC samples were TWIST1, TWIST2, ZEB1, ZEB2, and VIM (Figure 3).

After GEPIA, the mirDIP prediction tool was used to identify the level of interaction between the computationally selected miRNAs strongly involved in BC development and the main genes involved in EMT.

The mirDIP analysis showed that all 49 deregulated miRNAs in BC were able to interact with the 10 genes previously analyzed.

**Figure 4:**
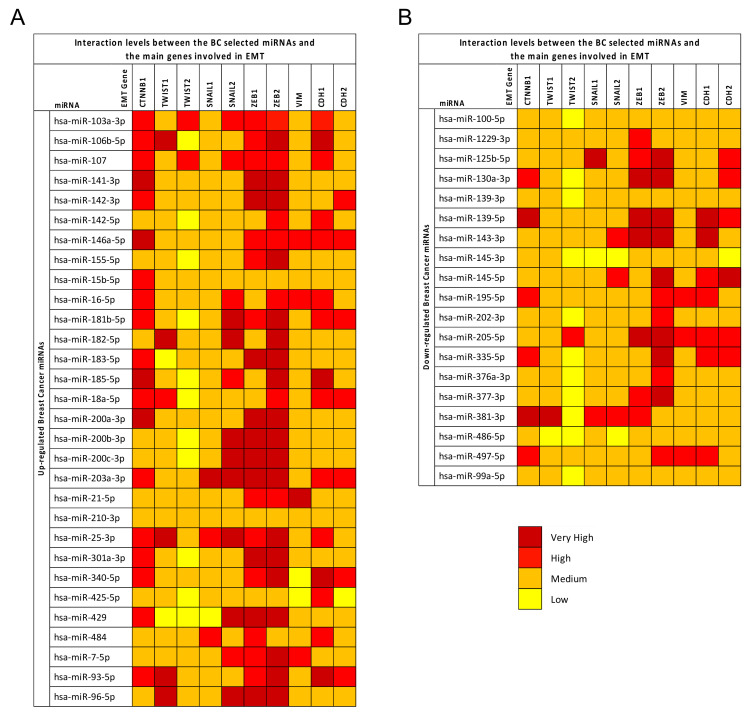
mirDIP analysis of BC miRNAs and EMT genes. Panel (**A**): Interaction between BC upregulated miRNAs and EMT genes. Panel (**B**): Interaction between BC downregulated miRNAs and EMT gene. The intensity of miRNA–gene interactions is reported as a red color scale ranging from yellow (low interaction levels) and dark red (very high interaction levels). Each EMT gene is reported with the level of interaction with the computationally selected BC miRNAs.

As reported in Figure 4, all selected miRNAs were able to target the EMT genes with interaction levels ranging from low to very high. Some genes were shown to be strongly modulated by the upregulated miRNAs. In particular, the CTNNB1, SNAIL2, ZEB1, and ZEB2 EMT genes showed the highest interaction levels with the upregulated miRNAs; in addition, ZEB1 and ZEB2 also showed very high interaction levels with some of the downregulated miRNAs. Generally, the upregulated miRNAs showed higher interaction levels with the EMT genes compared to the downregulated miRNAs. Of these miRNAs, the upregulated hsa-miR-106b-5p, hsa-miR-146a-5p, hsa-miR-203a-5p, and hsa-miR-25-3p showed very high interaction levels with almost all the EMT genes analyzed, suggesting that the modulation of these miRNAs may play a fundamental role in the regulation of cancer progression and in the development of the EMT phenotype. In contrast, the TWIST1 and TWIST2 genes showed the lowest interaction levels with all miRNAs (Figure 4).

**Figure 5:**
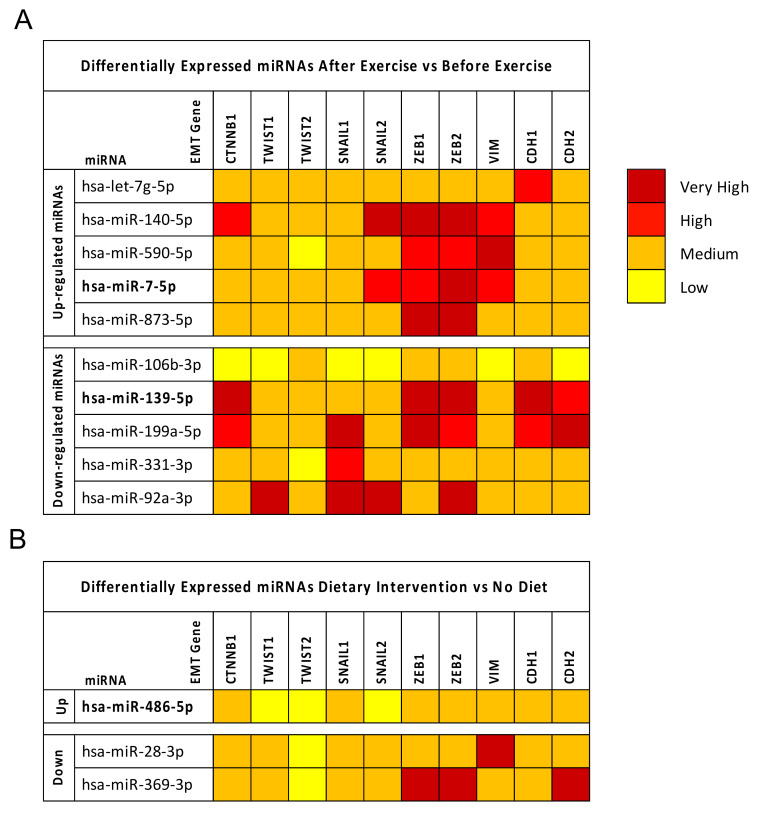
Panel (**A**): Interaction levels between EMT genes and miRNAs modulated by exercise. Panel (**B**): Interaction levels between EMT genes and miRNAs modulated by diet. The intensity of miRNA–gene interactions is reported as a red color scale ranging from yellow (low interaction levels) and dark red (very high interaction levels). For each EMT gene is reported the level of interaction with the computationally selected BC miRNAs.

The same mirDIP analysis on EMT genes was performed for the miRNAs selected by analyzing the diet and exercise datasets. Furthermore these miRNAs, particularly those obtained from the exercise datasets, were able to highly interact with the aforementioned EMT genes such as SNAIL2, ZEB1, and ZEB2. The most evident interactions were mediated by the upregulated hsa-miR-140-5p and the downregulated hsa-miR-139-5p, hsa-miR-199a-5p, and hsa-miR-92a-3p (Figure 5).

Contrary to what was observed for BC miRNAs, in this case, the highest interactions were found between downregulated miRNAs and EMT genes, especially with regard to diet-modulated miRNAs. Therefore, these results suggest that lifestyle interventions, and in particular dietary interventions, may represent a good therapeutic strategy aimed at reducing cancer aggressiveness, thus limiting the mesenchymal transformation of cancer cells.

### 2.5. microRNA Pathway Prediction Analysis and miRNA-Targeted Genes Protein–Protein Interaction

The DIANA-mirPath analysis performed on the 10 miRNAs modulated by exercise showed that, globally, these miRNAs were able to alter 59 different molecular and signaling transduction pathways deposited on Kyoto Encyclopedia of Genes and Genomes (KEGG) and involved in different cellular processes (data not shown). Specifically, these miRNAs were able to modulate 35 different pathways and 2006 genes directly and indirectly involved in cancer development and progression (Table 3).

Of these pathways, the most important and highly modulated were the following KEGG pathways: “Pathways in cancer (hsa05200)”, “Proteoglycans in cancer (hsa05205)”, “MAPK signaling pathway (hsa04010)”, and “PI3K-Akt signaling pathway (hsa04151)”. All these pathways are known to be altered in cancer including that of the breast. Overall, exercise is able to modulate 652 univocal genes through the modulation of the 10 computationally identified miRNAs. These results suggest that exercise may play a key role in counteracting cancer progression, in line with current evidence in vivo, and in support of the therapeutic efficacy of standard anticancer treatments. Indeed, among these genes, the most frequently altered within the 35 identified KEGG pathways were MAPK1 (27 out of 35 pathways), AKT1, AKT2, PIK3CA, PIK3CB, PIK3R3 (26 out of 35), AKT3, KRAS (23 out of 35), CCND1, TP53 (22 out of 35), HRAS, MAP2K2, NRAS, RAF1 (21 out of 35), BRAF (19 out of 35), and MYC (18 out of 35).

Overall, these miRNAs were potentially involved in the modulation of the MAPK family (including RAS/RAF/MAPK genes); Caspase family (strongly involved in apoptosis), Cyclin/CDK family; transcription factors; epidermal-, vascular endothelial-, fibroblast-, and platelet-derived growth factors; and the SMAD family, among others.

Regarding the three miRNAs directly modulated by diet, the DIANA-mirPath analysis revealed that diet-modulated miRNAs were able to modulate five different KEGG pathways: Adherens junction (hsa04520), Choline metabolism in cancer (hsa05231), Epithelial cell signaling in Helicobacter pylori infection (hsa05120), Colorectal cancer (hsa05210), and Glioma (hsa05214) (Table 4).

Overall, these miRNAs were able to modulate 44 genes of which 28 were univocal. Among these genes, the most frequently altered were EGFR and RAC1 (four out of five pathways), PLCG1 (three out of five), FOS, MET, NRAS, SMAD4, SOS2, TGFBR2, WASF1, and WASL (two out of five).

Although to a lesser extent than exercise, those miRNAs also directly modulated by diet were able to alter several KEGG pathways and, in turn, key tumor suppressors and oncogenes involved in cancer progression. In particular, diet-modulated miRNAs were able to target and interfere with several genes mainly involved in cancer signal transduction pathways (e.g., EGFR, RAC1, PLCG1, FOS, NRAS, SOS2, etc.).

**Figure 6:**
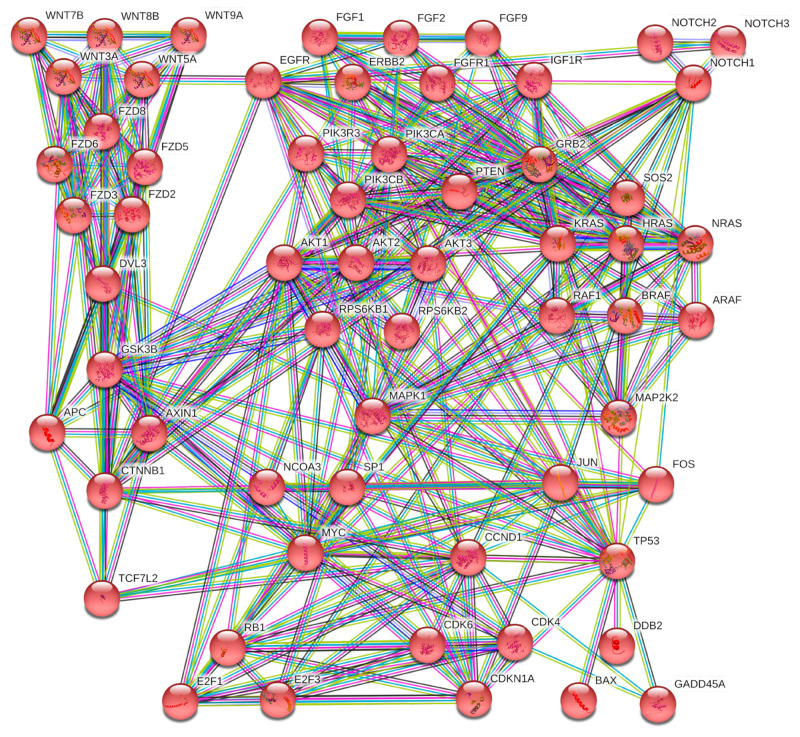
STRING protein interaction network of 61 out of 652 genes targeted by the selected miRNAs and directly involved in the breast cancer KEGG pathway (hsa05224).

To further corroborate these DIANA-mirPath results, Search Tool for the Retrieval of Interacting Genes/Proteins (STRING) and Gene Ontology (GO) analyses were performed to cluster the 652 univocal genes targeted by exercise-modulated miRNAs. In the STRING analysis, the high number of genes identified does not permit a clear clustering of all hubs and networks among genes to be obtained. Nevertheless, it showed that those genes were involved in 203 pathways and, in particular, 61 of them were strongly involved in the breast cancer KEGG pathway (hsa05224) (Figure 6).

In Figure 6, it is possible to note that the exercise-modulated miRNAs were able to modulate genes, and consequently proteins, at all levels of a signal transduction pathway. Overall, the computationally selected miRNAs were able to significantly alter the Wnt/B-catenin pathway, the MAPKs pathway, and the PI3K/Akt pathway by altering ligands (FGF and WNT families), receptors (EGFR, ERBB2, FGFR1, IGFR1, NOTCH family, and FZD receptors), intracellular tyrosine kinases (PI3K family, AKT family, RAS and RAF family, etc.), transcription factors (E2F family), and finally, effectors.

**Figure 7:**
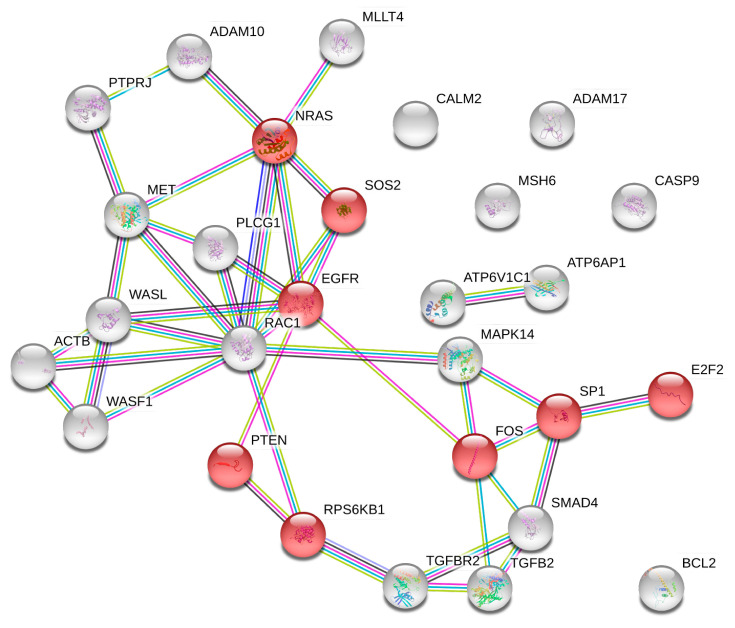
STRING interaction network between genes targeted by the diet-modulated miRNAs. In red are the genes involved in the breast cancer KEGG pathway (hsa05224).

The same STRING analysis was performed for the 28 univocal genes altered by diet-modulated miRNAs. In this case, STRING protein–protein interactions showed that the 28 proteins derived from the selected genes were able to alter 125 different KEGG pathways. Eight out of 28 proteins were also involved in the breast cancer pathway (hsa05224) and these proteins refer to EGFR and RAS signal transduction pathways, thus including proteins like EGFR, NRAS, PTEN, RPS6KB1, SP1, SOS2, FOS, and E2F2 transcription factor (Figure 7).

By merging the 652 exercise-modulated and the 28 diet-modulated genes, it was possible to identify 17 genes modulated by both diet and exercise and involved in several cancer pathways including that of BC.

In order to establish the functional role of the 652 exercise-modulated and the 28 diet-modulated genes, GO Panther analyses were performed to cluster such genes according to their Molecular Function, Biological Process, and Cellular Component.

**Figure 8:**
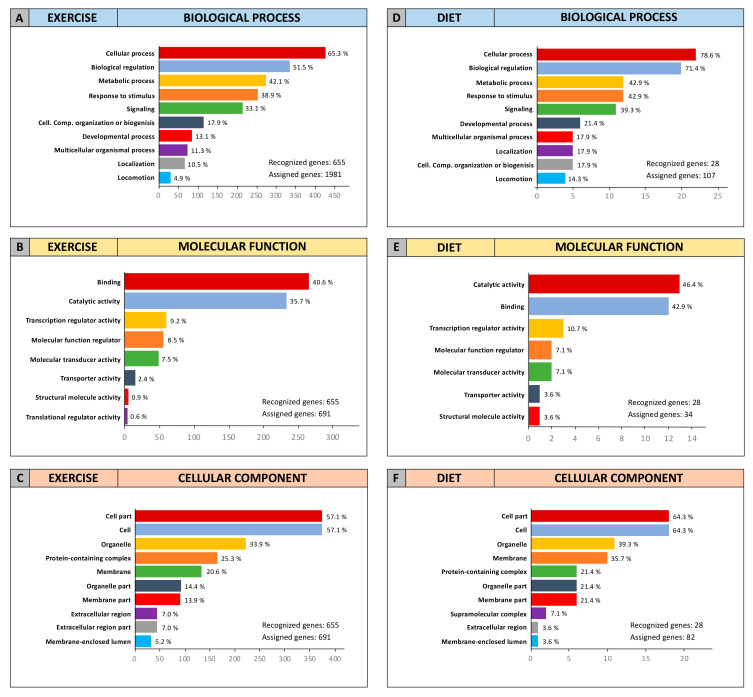
STRING and Gene Ontology (GO) enrichment analyses of exercise- and diet-miRNA-modulated genes. (**A**) Exercise-modulated genes clustered according to biological process; (**B**) exercise-modulated genes clustered according to molecular function; (**C**) exercise-modulated genes clustered according to cellular component; (**D**) diet-modulated genes clustered according to biological process; (**E**) diet-modulated genes clustered according to molecular function; and (**F**) diet-modulated genes clustered according to cellular component.

The two independent GO Panther analyses performed on exercise- and diet-modulated genes showed that, overall, the genes identified were involved in the same molecular functions and biological processes and were part of the same cellular component (Figure 8).

As shown in Figure 8, for the “Biological Process” category, the majority of exercise- and diet-modulated genes were involved in cellular process, biological regulation, metabolic process, response to stimulus, and in cellular signaling (Figure 8A,D). Concordant results were also obtained for the “Molecular Function” category, showing that almost 90% of exercise- and diet-modulated genes were involved in binding and catalytic activities as well as, to a lesser extent, transcriptional regulation (Figure 8B,E). Finally, the analysis of the “Cellular Component” category showed that the computationally identified genes were all part of the cell compartment, especially the cell, organelle, and membrane. However, genes also belonging to the extracellular region including ligands and growth factors were represented within both exercise- and diet-modulated genes (Figure 8C,F).

Overall, STRING and GO Panther analyses revealed that the miRNAs modulated by both diet and exercise induced the strong modulation of the main pathways and genes involved in key cellular processes responsible for BC development and therapeutic failure. Therefore, these encouraging results suggest how a healthy lifestyle and diet may positively influence the outcome of breast cancer patients after surgery.

### 2.6. Overall Survival Predictive Value of BC, Exercise-Modulated, and Diet-Modulated microRNAs

The OncoLnc analysis performed on the 49 BC miRNAs, the 10 exercise-modulated miRNAs, and the three diet-modulated miRNAs allowed for the identification of a miRNAs signature able to predict the prognosis of BC patients with respect to OS, taking into account the survival data contained in TCGA BRCA database.

**Figure 9:**
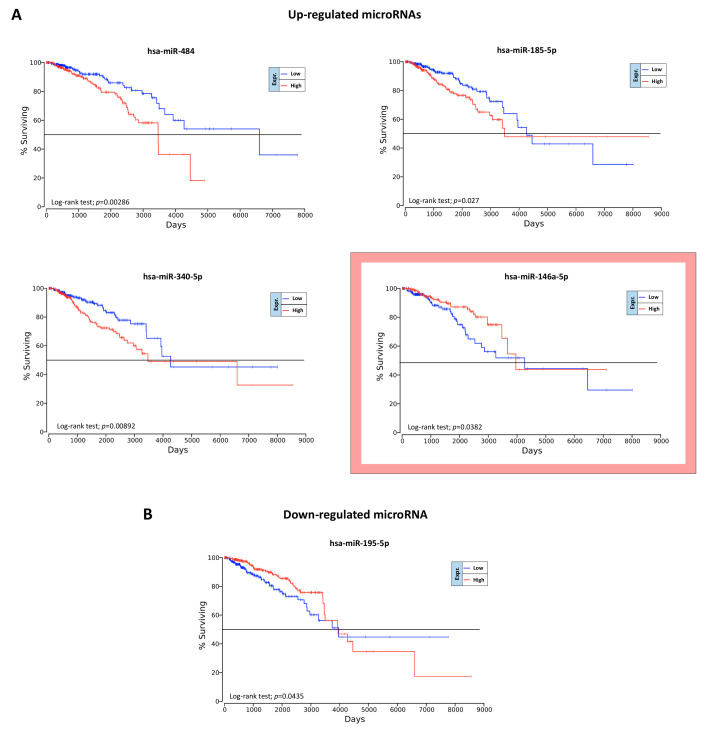
Prognostic value of BC dysregulated miRNAs according to OncoLnc. Panel (**A**): Upregulated miRNAs statistically associated with the survival of patients. In the red box is reported the upregulated miRNA whose expression is not concordant with survival curves. Panel (**B**): downregulated BC miRNA statistically associated with the survival of patients.

The OncoLnc analysis performed on the 49 BC miRNAs revealed that only five miRNAs were statistically associated with a lower OS (log-rank test, *p* < 0.05). Of these miRNAs, four were upregulated (hsa-miR-484, hsa-miR-185-5p, hsa-miR-340-5p, and hsa-miR-146a-5p) and only one, the hsa-miR-195-5p, was downregulated (Figure 9).

As shown in Figure 9A, one of the four upregulated miRNAs, the miRNA hsa-miR-146a-5p showed ambiguous results. Despite this miRNA being over-expressed in BC samples compared to controls, its upregulation was associated with a better prognosis (Figure 9A).

**Figure 10:**
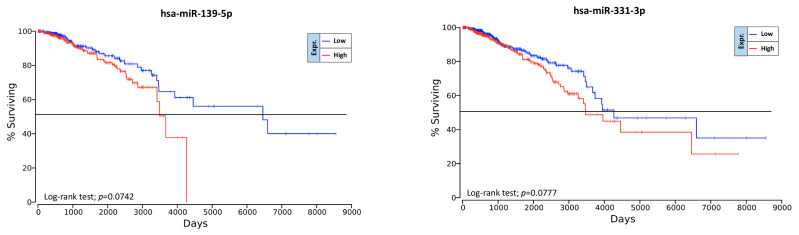
Prognostic value of exercise-modulated miRNAs according to OncoLnc.

Contrary to what was observed for the BC-associated miRNAs, no evidence was shown for the possible prognostic value of exercise- and diet-modulated miRNAs. In particular, none of the computationally selected miRNAs showed statistical significance with respect to OS. However, some miRNAs downregulated by exercise showed a weak association with the survival of BC patients. In particular, hsa-miR-139-5p and hsa-miR-331-3p downregulated by exercise showed interesting results (Figure 10). Indeed, although not statistically significant, these two downregulated miRNAs were associated with a better prognosis for BC patients. Therefore, an integrated treatment including exercise and/or diet that is able to downregulate these miRNAs may lead to a better prognosis for BC patients.

## 3. Discussion

It is known that environmental and lifestyle factors influence the health status of individuals. It has been widely demonstrated that healthy diets and physical activity have beneficial effects in several pathological conditions including cancer [55,56]. Several studies have highlighted how some foods with a high glycemic index or inflammatory index are associated with chronic inflammation, and in turn, to a plethora of inflammatory-related diseases including cancer, diabetes, and cardiovascular diseases [57,58]. Indeed, nutrients can directly or indirectly modulate the molecular and physiological mechanisms responsible for cell proliferation, differentiation, and programmed cell death. Some foods including red meat, sugar, and saturated fats may induce inflammation and oxidative stress, thereby predisposing cells to genetic mutations [59]. In contrast, other foods and nutrients including vegetables, fruit, olive oil, and prebiotics may prevent cancer development, possibly due to their high content of vitamins, minerals, bioflavonoids, and isoflavones, which have antioxidant and hormone-modulating properties, dietary fiber, which can beneficially affect human microbiota, and unsaturated fats involved in reduced inflammation [18,60,61]. Although some molecular mechanisms have been elucidated by which diet and exercise prevent cancer development and progression, to date there are still no clinical cancer studies on the effect of integrative treatments on epigenetic modifications. Accordingly, the value of epigenetic alterations in predicting the efficacy of integrative treatments and the prognosis of patients is not established yet.

In this context, our research group proposed a cross-sectional randomized clinical trial, the “Vitamin D, Exercise, Diet and Breast Cancer” study (DEDiCa) (NCT02786875), aimed at reducing the risk of BC recurrence and at increasing the disease-free survival (DFS) in women with surgically resected BC through the administration of vitamin D and healthy lifestyle including mild/moderate physical activity and a low glycemic index Mediterranean diet. A secondary aim of the DEDiCa study was to investigate whether changes in lifestyle may induce changes in the expression levels of miRNAs associated with BC characteristics and with patient prognosis [37].

In line with the DEDiCa study, the aim of the present study was to computationally identify miRNAs strictly associated with BC progression and with diet and exercise interventions. For this purpose, a series of rigorous bioinformatic analyses were performed in order to select miRNAs with diagnostic and prognostic significance for BC and to identify the molecular pathways and genes modulated by diet and exercise.

As demonstrated in different studies, the availability of several cancer databases and of a huge amount of bioinformatic data on tumor molecular and clinical features has allowed for the accurate identification of useful biomarkers for the early diagnosis of malignant tumors or to predict the risk of cancer relapse [62,63,64,65,66].

In the present study, we proposed the integrated analysis of different miRNA expression profiling datasets obtained from the GEO DataSets related to BC patients with dietary or exercise interventions. This approach allowed for the collection of miRNA expression data related to more than 1000 BC samples and healthy controls, which were merged with miRNA expression data obtained from more than 300 samples related to individuals undergoing diet or exercise interventions. The integration of different datasets and of a large number of samples allowed us to perform a more robust differential analysis compared to the analysis of single datasets. Furthermore, the selection of highly dysregulated miRNAs with concordant expression levels in the various datasets in which they were expressed allowed for the selection of those miRNAs actually involved in the development and progression of BC and certainly modulated by diet and exercise.

In particular, the integrated analysis of eight different BC miRNA microarray datasets allowed for the identification of a set of 49 upregulated and downregulated miRNAs in BC. Among these miRNAs, there were miRNAs already associated with the development of different tumors including those for BC. The most represented upregulated miRNAs were hsa-miR-106b-5p, hsa-miR-200a-3p, hsa-miR-21-5p, hsa-miR-141-3p, and hsa-miR-96-5p (over-expressed in seven out of eight datasets), while the most upregulated miRNAs (with higher log2FC value) were hsa-miR-182-5p, hsa-miR-183-5p, hsa-miR-7-5p, and the aforementioned hsa-miR-21-5p and hsa-miR-96-5p. Analogously, the most frequent downregulated miRNAs were hsa-miR-125b-5p and hsa-miR-99a-5p (downregulated in all eight analyzed datasets), hsa-miR-130a-3p, hsa-miR-205-5p, and hsa-miR-497-5p (seven out of eight datasets). Among the downregulated miRNAs, the higher log2FC were observed for hsa-miR-125b-5p, hsa-miR-139-5p, and hsa-miR-205-5p. The dysregulation of the identified miRNAs through the analysis of BC datasets was further confirmed by analyzing the miRNA expression data contained in TCGA BRCA database, thus corroborating the functional and diagnostic roles of these specific miRNAs in BC patients.

Of note, these dysregulated miRNAs are the result of the analysis of different datasets containing different molecular subtypes of BC. Therefore, the analysis of a single miRNA may not give useful results for the effective detection of BC, while the concomitant evaluation of 49 miRNAs is representative and potentially predictive for the identification of all BC subtypes.

Regarding the upregulated miRNAs identified, several studies have already highlighted their oncogenic role. For example, increased levels of the miR-200 family were associated with a worse prognosis in several cancers including that of the breast [67]. Similarly, hsa-miR-21-5p is considered one of the main oncogenic miRNAs and its upregulation is observed in several cancers (e.g., breast, colorectal cancer, pancreatic, and non-small cell lung cancers) [68,69]. Furthermore, a specific axis involving the upregulated miRNA hsa-miR-96-5p, hsa-miR-182-5p, and hsa-miR-183-5p were identified in breast cancer and other tumors and these miRNAs are known to be responsible for abnormal cell proliferation and migration [70,71,72].

Regarding the downregulated miRNAs, hsa-miR-125b-5p is recognized as the most common downregulated miRNA in several cancers [73]. Other identified miRNAs like hsa-miR-139-5p, hsa-miR-205-5p, hsa-miR-486-5p, and hsa-miR-99a-5p have shown strong involvement in neoplastic transformations and some studies are trying to use these miRNAs for the development of novel therapeutic strategies in BC [74,75,76,77]. This evidence corroborates the real diagnostic and prognostic significance of these miRNAs in patients affected by BC. Therefore, the set of miRNAs we identified may be proposed as a panel of miRNAs for the monitoring of patients with BC. However, a single miRNA cannot be univocally considered as a tumor suppressor or oncomiR. Indeed, each miRNA is able to modulate the expression levels of different genes including both oncogenes and tumor suppressor genes. Therefore, the tumor-promoting or suppressive action of miRNAs is the result of a complex network of interactions that is established between different miRNAs and different genes involved in neoplastic transformation.

Although several studies have confirmed the specificity of selected miRNAs in BC, there are no concordant studies on the possible miRNA modulating effect of diet and exercise, especially for those miRNAs involved in BC. In this context, our results showed that 10 miRNAs were selectively modulated by exercise interventions and three by diet. Among those miRNAs dysregulated in BC, hsa-miR-7-5p, hsa-miR-139-5p, and hsa-miR-486-5p were also significantly modulated by exercise and diet. Therefore, the evaluation of these three miRNAs will be useful not only to predict the prognosis of patients, but also to evaluate the effectiveness of the integrative treatments as well as the adherence of patients to the dietary and exercise recommendations. Aside from these three miRNAs strongly associated with BC, the other miRNAs modulated by diet and exercise may also play a fundamental role in BC; of these miRNAs, the upregulated miRNA hsa-miR-873-5p and the downregulated miRNA hsa-miR-199a-5p are involved in BC cells stemness and in tumor suppression, respectively [78,79].

The importance of the diet- and exercise-modulated miRNAs here identified for BC patients was further highlighted by the GEPIA and mirDIP analyses performed on the most important EMT genes. First, the GEPIA analysis showed that the expression levels of the epidermal marker CDH1 were upregulated in patients with BC compared to healthy individuals; in contrast, the mesenchymal marker (VIM) and the genes promoting the mesenchymal transition (SNAIL1/2, ZEB1/2, and TWIST1/2) were all downregulated in BC samples compared to the controls. These trends of expression are typical of all tumors originating from the epithelia. Subsequently, the mirDIP analysis revealed that both BC miRNAs and diet- and exercise-modulated miRNAs were able to strongly target the EMT genes whose dysregulation is associated with a worse prognosis in BC. In particular, both BC and diet- and exercise- differentially expressed miRNAs were able to strongly interact with genes strongly responsible for mesenchymal transition including CTNNB1, ZEB1/2, and SNAIL2, suggesting that the fine regulation of such miRNAs mediated by dietary and exercise interventions may prevent EMT, thus reducing the risk of BC recurrence [80]. To the best of our knowledge, for the first time, the interactions between diet- and exercise-modulated miRNAs and genes potentially involved in BC were here investigated, thus suggesting a relationship between diet, exercise, BC, and epigenetic mechanisms.

As a further demonstration of the effective involvement of both BC and diet- and exercise-modulated miRNAs in BC, the DIANA-mirPath analysis showed that the selected miRNAs were able to alter a total of 38 different cancer-related KEGG pathways (35 altered by BC miRNAs and five by diet- and exercise-modulated miRNAs; of these latter pathways, two were in common with the previous 35 pathways). Within these pathways, the most targeted genes were genes belonging to the Wnt/B-catenin pathway, the MAPKs pathway, and the PI3K/Akt pathway such as MAPK1, AKT family, PIK3 family, RAS/RAF genes, CCND1, EGFR, PTEN, etc. The alteration of all of these genes is known to be responsible for the development of BC [81]. In particular, the alteration of the PI3K/AKT pathway is recognized as a hallmark of BC. Recent studies have highlighted how the modulation of some regulatory proteins in the PI3K/AKT pathway is mediated by various miRNAs [82,83]. In the same manner, several studies have demonstrated that the dysregulation of CCND1 is associated with cancer progression while the over-expression of EGFR and EGFR-ligands as well as the dysregulation of the RAS/RAF/ERK signaling pathway are associated with the acquisition of drug resistance associated with cancer progression [84,85,86].

The involvement of these miRNA-targeted genes in BC was not only predicted, but also validated through the use of STRING and GO Panther enrichment analyses. These analyses thus confirmed that these genes are strongly involved in the breast cancer KEGG pathways (hsa05224), affecting different biological process and molecular functions as well as all cellular levels from the extracellular portion with the modulation of signal molecules (WNT and FGF families) up to the nucleus with the modulation of different transcription factors (E2F family) [87].

Finally, other important findings derived from the OncoLnc analysis allowed for the identification of miRNAs predictive of patient outcome.

Although the OncoLnc analysis performed on diet- and exercise- modulated miRNAs did not produce statistically significant results, the same analysis conducted on those 49 miRNAs closely associated with the development of BC revealed five miRNAs predicting overall survival (i.e., the downregulated miRNAs hsa-miR-484, hsa-miR-185-5p, hsa-miR-340-5p, and hsa-miR-146a-5p and the upregulated miRNA hsa-miR-195-5p). Several researchers have attempted to independently validate the prognostic significance of these miRNAs, albeit with controversial results [88,89,90,91,92]. However, here we suggest a novel prognostic strategy for the management of BC based on the evaluation of multiple miRNAs instead of single miRNAs that alone are not predictive of BC recurrence or patient survival.

Interestingly, although not statistically significant, the hsa-miR-139-5p and hsa-miR-331-3p downregulated by exercise may be good indicators of the efficacy of the integrative intervention and of patient prognosis as demonstrated by two independent studies [93,94].

Overall, the bioinformatic analyses described above allowed for the identification of specific miRNAs able to predict the risk of BC recurrence, the efficacy of the diet and exercise treatments, and patient prognosis. These findings, together with the new diagnostic approaches based on liquid biopsy samples, circulating tumor DNA (ctDNA) [95], and high-sensitive techniques like droplet digital PCR (ddPCR) [96,97,98,99], will allow for the effective validation of the prognostic significance of selected miRNAs in liquid biopsy samples obtained from BC patients treated with integrative interventions in order to improve current follow-up strategies as well as their quality of life.

However, as previously stated, the identification of promising non-invasive biomarkers cannot be based on the analysis of a single miRNA, but it is necessary to carry out the integrated analysis of a panel of miRNAs or different circulating markers (including miRNAs, proteins, and genetic or expression markers) to increase the specificity of liquid biopsy-based diagnostic strategies. In addition, it is important to note that often inconsistency exists between the expression levels of miRNA in tissue and liquid biopsy samples [100]. Therefore, the analysis of liquid biopsy samples may need to be confirmed by the analysis of miRNA expression in tissue specimens.

## 4. Materials and Methods

### 4.1. microRNA Expression Profiling Dataset Selection

For the identification of miRNAs strictly involved in the development and progression of BC and actively modulated by diet and exercise, the publicly available GEO DataSets database was used. Three different searches were performed in order to select microarray miRNA expression profiling datasets related to BC, dietary, and exercise interventions, respectively. For this purpose, three independent advanced searches were performed as previously described [101]. In particular, for the selection of microarray datasets containing data about miRNA expression levels in BC, the following search terms were used: “((breast cancer) AND ‘non coding rna profiling by array’ [DataSet Type]) AND ‘Homo sapiens’ [porgn:__txid9606]”.

For the selection of microarray expression profiling datasets related to dietary and exercise interventions, the following two search strings were used: “((diet) AND ‘non coding rna profiling by array’ [DataSet Type]) AND ‘Homo sapiens’ [porgn:__txid9606]” and “((Exercise) AND ‘non coding rna profiling by array’ [DataSet Type]) AND ‘Homo sapiens’ [porgn:__txid9606]”.

All three searches were performed on the relevant datasets available up to March 2020.

Regarding the datasets selected for diet and exercise, the only inclusion criterion adopted was datasets containing miRNA expression levels of treated and untreated human samples, thus discarding all datasets obtained from animal models, cell lines, or other in vitro experiments.

Instead, regarding the selection of BC miRNA datasets, the advanced search allowed for the identification of a list of all BC datasets containing miRNA expression levels. The inclusion and exclusion criteria for these datasets were as follows:

Inclusion criteria: (i) datasets containing miRNA expression levels of BC tissues excluding datasets containing serum or plasma samples; (ii) datasets reporting miRNAs expression levels of both tumor and normal tissue samples; and (iii) datasets containing miRNA expression data of at least 10 tumor samples and 10 controls.

Exclusion criteria: (i) datasets containing exclusively tumor samples; (ii) datasets containing miRNAs expression levels of animal models, cell lines or other in vitro experiments; and (iii) datasets containing miRNAs expression levels detected in serum/plasma samples.

In particular, data derived from liquid biopsy samples were discarded in order to select only datasets containing tissue samples with miRNA expression levels strictly associated with the tumor mass.

Finally, the miRNA expression data contained in the “miRNA mature strand expression RNAseq” dataset of the Cancer Genome Atlas Breast Cancer (TCGA BRCA) database were analyzed to further confirm the miRNA expression results obtained from GEO DataSets microarray platforms.

### 4.2. Differential Analysis between Groups and microRNA Annotation

Regarding the BC datasets, differential analyses were performed between the tumor samples and normal breast tissues, while for the diet and exercise datasets, the differential analyses of miRNAs expression levels were performed between dietary and exercise treated and untreated samples.

In particular, the data matrices were obtained from each GSE dataset and samples were stratified into two distinctive groups (Tumors vs. Normal; Diet vs. No Diet, Exercise vs. No Exercise). Before differential analysis, the miRNAs of each dataset were annotated using the latest version of miRBase nomenclature (V.22.) (http://www.mirbase.org/), thus converting the sequence or the miRNA ID (MIMAT00 code) of each miRNA in the following nomenclature “hsa-miR-”. The differential analyses between groups were subsequently performed by using the GEO2R software publicly available on GEO DataSets. The results of the differential analyses were expressed as base-2 logarithm of Fold Change (log2FC) in order to normalize the variability of results obtained from different microarray technologies.

miRNAs with log2FC with a statistical significance of *p* < 0.01 (for BC datasets) or *p* < 0.05 (for diet and exercise datasets) were selected as significantly deregulated miRNAs.

### 4.3. Identification of microRNAs Involved in Breast Cancer and Effectively Modulated by Diet and Exercise

The lists of differentially expressed miRNAs obtained for each BC dataset were subsequently merged by using the publicly available Venn Diagrams of the Bioinformatics & Evolutionary Genomics (BEG) tool (http://bioinformatics.psb.ugent.be/webtools/Venn/). The same analysis was performed for the diet and exercise datasets.

After data merging, among all the dysregulated miRNAs in the BC, diet, and exercise datasets, only those highly upregulated or downregulated and with concordant expression levels in more than 50% of the analyzed datasets were selected.

For each miRNA, the log2FC was reported indicating with red boxes the over-expressed miRNAs and with blue boxes the downregulated ones.

### 4.4. Interaction between Selected microRNAs and Epithelial-Mesenchymal Transition (EMT) Genes

After miRNA selection, the basal expression levels of EMT genes including CTNNB1, TWIST1/2, SNAIL1/2, ZEB1/2, VIM, CDH1 (E-Cad), and CDH2 (N-Cad) were analyzed in the BC samples and in healthy controls. For this purpose, the GEPIA software (http://gepia.cancer-pku.cn/index.html), capable of deriving and processing the RNA sequencing expression data of 1085 breast cancer samples and 291 normal breast tissues contained in the TCGA Breast Invasive Carcinoma and GTEx datasets, was used [102].

Subsequently, the interaction levels between the computationally identified BC miRNAs and genes responsible for EMT were established by using the bioinformatic tool microRNA Data Integration Portal (mirDIP—Version 4.1.11.1, Database version 4.1.0.3, September 2018) (http://ophid.utoronto.ca/mirDIP). In particular, mirDIP software allowed for the integration of the human RNA-gene target predictions contained in 30 different miRNA prediction databases, thus obtaining more robust data about the interaction levels between miRNAs and gene targets and avoiding database-specific bias [103]. The mirDIP analysis was performed for the aforementioned EMT genes: CTNNB1, TWIST1/2, SNAIL1/2, ZEB1/2, VIM, CDH1 (E-Cad) and CDH2 (N-Cad).

### 4.5. microRNA Pathway Prediction Analysis

For the diet- and exercise-modulated miRNAs previously identified, a pathway prediction analysis was performed to investigate the role of such miRNAs in the modulation of molecular and signal transduction pathways known to be involved in tumor progression. For this purpose, the computational prediction tool DIANA-mirPath (v.3) was used as previously described [104]. Briefly, DIANA-mirPath is able to identify the molecular pathways significantly modulated by a list of miRNAs predicting the targeted 3′-UTR gene regions by consulting the experimentally validated miRNA interactions obtained from the DIANA-TarBase v7.0 database [105]. These interactions (predicted and/or validated) were subsequently combined with sophisticated merging and meta-analysis algorithms by DIANA-mirPath, giving as a result the genes and pathways targeted by a specific miRNA and the statistical significance of this interaction.

### 4.6. miRNA-Targeted Genes Interaction and Gene Ontology (GO)

To better understand the functional effects of exercise- and diet- modulated miRNAs, STRING: Functional protein association networks’ (https://string-db.org/) software was used to establish the interaction network of the genes identified through DIANA-mirPath analysis [106]. For both exercise- and diet- modulated genes, the involvement within the breast cancer KEGG pathway (hsa05224) was highlighted.

For the genes identified through DIANA-mirPath, the software GO Panther (GO Panther v.14.0—http://pantherdb.org/) [107] was used to perform GO analysis. The DIANA-mirPath-selected genes were classified according to their Molecular Function (MC), Biological Process (BP), and Cellular Component (CC).

### 4.7. Prognostic Significance of Computationally Selected microRNAs

In order to determine the prognostic significance of the computationally selected miRNAs obtained from the BC, diet, and exercise datasets, the bioinformatics tool OncoLnc (http://www.oncolnc.org/) was used [108]. In particular, OncoLnc is able to calculate Kaplan–Meier curves by analyzing the miRNA expression and survival data derived from TCGA BRCA datasets. The Kaplan–Meier curves were obtained following the instruction provided by the developers of the software, thus comparing the miRNA expression levels and survival data of the bottom quartile samples and top quartile samples. Overall survival (OS) curves were reported only when the log-rank *p*-value was *p* < 0.05.

### 4.8. Statistical Analyses

The log2FC values of the computationally selected miRNAs were already normalized by the GEO2R software. As stated before, for the BC datasets, miRNAs with a *p*-value of *p* < 0.01 were selected, while for the diet and exercise datasets, miRNAs with a *p*-value of *p* < 0.05 were selected. With regard to the GO and pathway prediction analyses, STRING, GO Panther, and DIANA-mirPath automatically perform proper statistical tests, providing statistically significant data expressed as *p*-values. Finally, for the Kaplan–Meier analysis, only overall survival curves with a log-rank *p*-value *p* < 0.05 were selected.

## 5. Conclusions

The integration of different miRNA expression microarray datasets and the use of several bioinformatics prediction tools has allowed for the identification of miRNAs strictly involved in BC. Among these, the miRNAs hsa-miR-182-5p, hsa-miR-183-5p, hsa-miR-200 family, hsa-miR-21-5p, hsa-miR-7-5p, hsa-miR-96-5p, hsa-miR-125b-5p, hsa-miR-139-5p, hsa-miR-497-5p, hsa-miR-99a-5p, and hsa-miR-486-5p were altered in BC and modulated by diet and exercise interventions while the miRNAs hsa-miR-484, hsa-miR-185-5p, hsa-miR-340-5p, hsa-miR-146a-5p, hsa-miR-195-5p, hsa-miR-139-5p, and hsa-miR-331-3p were specific for the prediction of the survival of patients. These data suggest that diet and exercise exert a double protective role in cancer through the modulation of epigenetic factors and the well-documented positive regulation of gene expression.

The *in silico* results here obtained represent the starting point for the in vivo validation that will be performed in liquid biopsy samples collected from BC patients. In particular, future validation studies will benefit from our clinical trial DEDiCa, where the expression levels of miRNAs identified in the current *in silico* study will be analyzed in liquid biopsy samples obtained from BC patients treated with a lifestyle treatment by using specific ddPCR probes for the selected miRNAs. The miRNA expression results will be further compared with the clinical-pathological features of patients and the data obtained from food and physical activity diaries in order to establish the effectiveness of DEDiCa treatment in reducing the risk of BC recurrence and in improving the general health and quality of life of patients.

## Figures and Tables

**Table 1:** Main characteristics of the selected datasets.

Series Accession	Number of Control	Number of BC Tissue	Total Number	Samples	Platform	Ref.
				Breast Cancer Datasets		
GSE57897	31	422	453	Normal and BC tissues	GPL18722 Homo sapiens microRNA array	[38]
GSE97811	16	45	61	Normal and BC FFPE tissues	GPL21263 3D-Gene Human miRNA V21_1.0.0	[39]
GSE58606	11	122	133	Normal and BC FFPE tissues	GPL18838 miRCURY LNA microRNA Array 7th generation	[40]
GSE38167	23	31	54	Normal and BC FFPE tissues	GPL14943 Agilent-029297 Human miRNA Microarray	[41]
GSE32922	15	22	37	Normal and BC tissues	GPL7723 miRCURY LNA microRNA Array, v.11.0	[42]
GSE45666	15	101	116	Normal and BC tissues	GPL14767 Agilent-021827 Human miRNA Microarray	[43]
GSE26666	17	77	94	Frozen normal and BC tissues	GPL8227 Agilent-019118 Human miRNA Microarray 2.0	[44]
GSE40525	59	61	120	Normal and BC FFPE tissues	GPL8227 Agilent-019118 Human miRNA Microarray 2.0	[45]
	**Samples before Diet**	**Samples after Diet**		**Diet Datasets**		
GSE27474	14	14	28	Serum Samples	GPL8179 Illumina Human v2 MicroRNA expression beadchip	[46]
GSE87103	12	12	24	Subcutaneous adipose tissues	GPL11434 miRCURY LNA microRNA Array, 6th generation	[47]
GSE75026	12	12	24	PBMCs samples	GPL8179 Illumina Human v2 MicroRNA expression beadchip	[48]
	**Samples before Exercise**	**Samples after Exercise**		**Exercise Datasets**		
GSE133910	46	44	90	Peripheral blood samples	GPL25134 Agilent-070156 Human_miRNA_V21.0_Microarray	[49]
GSE87103	12	12	24	Subcutaneous adipose tissues	GPL11434 miRCURY LNA microRNA Array, 6th generation	[47]
GSE45041	10	10	20	White blood cells	GPL16770 Agilent-031181 Unrestricted Human miRNA V16.0	[50]
GSE51837	12	12	24	Monocytes	GPL10850 Agilent-021827 Human miRNA Microarray (V3)	[51]
GSE41915	11	11	22	NK cells	GPL10850 Agilent-021827 Human miRNA Microarray (V3)	[52]
GSE28745	12	12	24	PBMCs	GPL8227 Agilent-019118 Human miRNA Microarray 2.0	[53]
GSE18999	11	11	22	Neutrophils	GPL7731 Agilent-019118 Human miRNA Microarray 2.0	[54]

**Table 2:** Validation of the GEO DataSets selected miRNAs in the Cancer Genome Atlas Breast Cancer database.

miRNA	GEO Datasets log2FC Average	TCGA BRCA	
			*p*-Value
**Upregulated miRNAs**			
hsa-miR-103a-3p	1.044	1.532	1.031 × 10^−19^
hsa-miR-106b-5p	1.000	2.423	7.270 × 10^−28^
hsa-miR-107	0.882	1.676	3.424 × 10^−17^
hsa-miR-141-3p	1.476	5.343	2.723 × 10^−29^
hsa-miR-142-3p	1.775	3.040	2.214 × 10^−18^
hsa-miR-142-5p	1.413	2.283	1.313 × 10^−13^
hsa-miR-146a-5p *	0.902	#N/D	#N/D
hsa-miR-155-5p	1.443	1.961	4.674 × 10^−18^
hsa-miR-15b-5p	1.076	1.908	1.745 × 10^−11^
hsa-miR-16-5p	0.955	1.591	2.960 × 10^−8^
hsa-miR-181b-5p	0.616	2.219	1.238 × 10^−22^
hsa-miR-182-5p	2.027	4.799	2.421 × 10^−40^
hsa-miR-183-5p	2.759	7.102	3.388 × 10^−47^
hsa-miR-185-5p	0.883	1.548	2.932 × 10^−9^
hsa-miR-18a-5p	1.714	1.905	5.766 × 10^−14^
hsa-miR-200a-3p	1.346	3.948	2.352 × 10^−26^
hsa-miR-200b-3p	1.564	2.846	4.468 × 10^−20^
hsa-miR-200c-3p	1.298	2.691	2.049 × 10^−17^
hsa-miR-203a-3p	1.055	2.287	2.680 × 10^−14^
hsa-miR-21-5p	2.054	4.448	5.680 × 10^−41^
hsa-miR-210-3p	1.244	5.632	1.111 × 10^−31^
hsa-miR-25-3p *	0.946	#N/D	#N/D
hsa-miR-301a-3p	1.620	2.314	7.058 × 10^−24^
hsa-miR-340-5p	0.924	2.001	8.384 × 10^−25^
hsa-miR-425-5p	1.733	1.740	8.393 × 10^−8^
hsa-miR-429	1.751	4.884	6.208 × 10^−33^
hsa-miR-484	0.792	1.399	2.801 × 10^−6^
hsa-miR-7-5p	1.786	1.850	7.483 × 10^−21^
hsa-miR-93-5p	1.209	2.110	5.223 × 10^−26^
hsa-miR-96-5p	2.178	6.970	7.515 × 10^−47^
**Downregulated miRNAs**			
hsa-miR-100-5p	−1.574	−4.066	1.191 × 10^−52^
hsa-miR-1229-3p	−0.819	1.274	2.105 × 10^−6^
hsa-miR-125b-5p	−1.819	−4.107	6.680 × 10^−48^
hsa-miR-130a-3p	−0.996	−2.371	2.085 × 10^−30^
hsa-miR-139-3p	−1.178	−8.512	2.131 × 10^−52^
hsa-miR-139-5p	−2.444	−8.699	6.482 × 10^−58^
hsa-miR-143-3p	−1.422	−2.288	1.458 × 10^−28^
hsa-miR-145-3p	−1.501	−3.196	7.255 × 10^−54^
hsa-miR-145-5p	−2.015	−5.892	2.501 × 10^−50^
hsa-miR-195-5p	−1.541	−2.867	1.560 × 10^−39^
hsa-miR-202-3p *	−1.198	#N/D	#N/D
hsa-miR-205-5p	−2.033	−5.921	4.198 × 10^−35^
hsa-miR-335-5p	−1.961	−4.846	4.980 × 10^−27^
hsa-miR-376a-3p	−1.441	−1.364	2.287 × 10^−8^
hsa-miR-377-3p	−1.542	−1.418	2.186 × 10^−7^
hsa-miR-381-3p	−1.523	−2.486	3.350 × 10^−26^
hsa-miR-486-5p	−1.496	−10.611	3.737 × 10^−26^
hsa-miR-497-5p	−1.254	−2.726	1.543 × 10^−33^
hsa-miR-99a-5p	−1.433	−5.165	6.270 × 10^−63^

* Non-significantly dysregulated miRNAs in the TCGA BRCA database; in bold miRNAs with discordant expression levels between the GEO DataSets and TCGA databases.

**Table 3:** Molecular and signaling transduction pathways altered by the 10 miRNAs modulated by exercise and involved in cancer.

KEGG Pathway	*p*-Value	N. of Targeted Genes	Involved miRNAs
Pathways in cancer (hsa05200)	4.05564 × 10^−7^	179	10
PI3K-Akt signaling pathway (hsa04151)	0.011123026	140	10
Proteoglycans in cancer (hsa05205)	2.21725 × 10^−17^	107	10
MAPK signaling pathway (hsa04010)	0.011123026	104	10
Viral carcinogenesis (hsa05203)	6.08511 × 10^−8^	95	10
Transcriptional misregulation in cancer (hsa05202)	0.002760238	80	10
Hippo signaling pathway (hsa04390)	1.31008 × 10^−8^	76	10
FoxO signaling pathway (hsa04068)	1.48491 × 10^−6^	71	10
Cell cycle (hsa04110)	3.14391 × 10^−5^	66	10
Insulin signaling pathway (hsa04910)	0.009388457	65	10
AMPK signaling pathway (hsa04152)	0.006097228	62	10
Thyroid hormone signaling pathway (hsa04919)	0.000182888	61	10
HIF-1 signaling pathway (hsa04066)	3.77879 × 10^−5^	57	10
TNF signaling pathway (hsa04668)	0.014299582	54	10
Prostate cancer (hsa05215)	2.74229 × 10^−5^	52	10
Small cell lung cancer (hsa05222)	2.1672 × 10^−5^	50	10
Estrogen signaling pathway (hsa04915)	0.00014031	47	10
ErbB signaling pathway (hsa04012)	0.001313884	45	9
Chronic myeloid leukemia (hsa05220)	1.45098 × 10^−6^	44	10
Renal cell carcinoma (hsa05211)	1.69642 × 10^−7^	42	10
TGF-beta signaling pathway (hsa04350)	4.62474 × 10^−6^	41	10
Pancreatic cancer (hsa05212)	9.61555 × 10^−6^	40	10
Colorectal cancer (hsa05210)	5.0244 × 10^−7^	39	10
p53 signaling pathway (hsa04115)	0.00014031	39	10
Apoptosis (hsa04210)	0.018765462	38	10
Glioma (hsa05214)	2.56338 × 10^−6^	37	10
Central carbon metabolism in cancer (hsa05230)	2.56338 × 10^−6^	36	10
Non-small cell lung cancer (hsa05223)	8.72499 × 10^−6^	34	10
ECM-receptor interaction (hsa04512)	1.96805 × 10^−5^	34	10
mTOR signaling pathway (hsa04150)	0.003253273	34	10
Melanoma (hsa05218)	0.009412459	34	10
Endometrial cancer (hsa05213)	3.18028 × 10^−5^	32	10
Acute myeloid leukemia (hsa05221)	0.019544711	29	10
Bladder cancer (hsa05219)	0.000423154	25	9
Thyroid cancer (hsa05216)	0.000804104	17	10

**Table 4:** KEGG pathways altered by the three miRNAs modulated by diet.

KEGG Pathway	*p*-Value	N. of Targeted Genes	Involved miRNAs
Adherens junction (hsa04520)	0.00127981	10	2
Choline metabolism in cancer (hsa05231)	0.0417592	10	2
Epithelial cell signaling in Helicobacter pylori infection (hsa05120)	0.04463459	9	3
Colorectal cancer (hsa05210)	0.02665458	8	2
Glioma (hsa05214)	0.02665458	7	3
